# The Effects of Air Pollution on Hospitalizations for Cardiovascular Disease in Elderly People in Australian and New Zealand Cities

**DOI:** 10.1289/ehp.8674

**Published:** 2006-03-13

**Authors:** Adrian G. Barnett, Gail M. Williams, Joel Schwartz, Trudi L. Best, Anne H. Neller, Anna L. Petroeschevsky, Rod W. Simpson

**Affiliations:** 1 School of Population Health, University of Queensland, Herston, Australia; 2 Exposure, Epidemiology, and Risk Program, Harvard School of Public Health, Harvard University, Boston, Massachusetts, USA; 3 Faculty of Science, Health and Education, University of the Sunshine Coast, Maroochydore, Australia

**Keywords:** air pollution, Australia, cardiovascular disease, meta-analysis, New Zealand

## Abstract

**Objective:**

The goal of this study was to estimate the associations between outdoor air pollution and cardiovascular hospital admissions for the elderly

**Design:**

Associations were assessed using the case–crossover method for seven cities: Auckland and Christchurch, New Zealand; and Brisbane, Canberra, Melbourne, Perth, and Sydney Australia. Results were combined across cities using a random-effects meta-analysis and stratified for two adult age groups: 15–64 years and ≥ 65 years of age (elderly). Pollutants considered were nitrogen dioxide, carbon monoxide, daily measures of particulate matter (PM) and ozone. Where multiple pollutant associations were found, a matched case–control analysis was used to identify the most consistent association.

**Results:**

In the elderly, all pollutants except O_3_ were significantly associated with five categories of cardiovascular disease admissions. No associations were found for arrhythmia and stroke. For a 0.9-ppm increase in CO, there were significant increases in elderly hospital admissions for total cardiovascular disease (2.2%), all cardiac disease (2.8%), cardiac failure (6.0%), ischemic heart disease (2.3%), and myocardial infarction (2.9%). There was some heterogeneity between cities, possibly due to differences in humidity and the percentage of elderly people. In matched analyses, CO had the most consistent association.

**Conclusions:**

The results suggest that air pollution arising from common emission sources for CO, NO_2_, and PM (e.g., motor vehicle exhausts) has significant associations with adult cardiovascular hospital admissions, especially in the elderly, at air pollution concentrations below normal health guidelines.

**Relevance to clinical and professional practice:**

Elderly populations in Australia need to be protected from air pollution arising from outdoor sources to reduce cardiovascular disease.

There have been several studies on the short-term effects of air pollution on hospital admissions (Burnett et al. 1997a, 1997b; Le Tertre et al. 2002; Pope 2000; Samet et al. 2000), but most have examined single cities. Such single-city studies have been criticized for being applicable only to the city under study and for using different modeling approaches. These comments have led to multicity meta-analyses where the results are pooled—for example, the National Morbidity, Mortality, and Air Pollution Study (NMMAPS) conducted on behalf of the Health Effects Institute in the United States and the APHEA (Air Pollution and Health: A European Approach) studies in Europe. NMMAPS examined the associations between daily hospital counts for cardiovascular admissions in the elderly and air pollutants in 14 cities in different regions of the United States (Dominici et al. 2002b; Samet et al. 2000). The APHEA studies have taken place in two stages, and the latest (APHEA2) comprised eight European cities in the investigation of associations of air pollution on daily cardiovascular admissions (Le Tertre et al. 2002). Multicity studies have also been conducted in Canada (Burnett et al. 1997a, 1997b).

Despite these studies, the strength of the association between outdoor air pollution and health effects is still unclear because of the complexity of the time-series modeling. In addition, when multiple pollutants have been examined, the independent effects of each pollutant are usually addressed in multipollutant models, but these are sensitive to the modeling assumptions. If the association with one pollutant is nonlinear or varies by season, then a two-pollutant model assuming a linear relationship with each pollutant might not give the independent effect of the second pollutant. Therefore, the case–crossover design (Maclure 1991), which is less sensitive to model assumptions, is more appropriate. This method investigates the effects of acute exposures and can also examine both multiple exposures and interactions between exposures. It has been applied to the analysis of the acute effects of environmental exposures, especially air pollution (Sunyer et al. 2000). The method matches case days to nearby control days and hence controls for covariates that change slowly over time (e.g., age, smoking behavior, and usual diet). Such matching also controls for seasonal variation and time trends in the health event (Bateson and Schwartz 2001).

In this study we aimed to find associations between outdoor air pollutant and cardiovascular disease (as measured by counts of hospital admissions) in cities in Australia and New Zealand. The study used two age groups, ≥ 65 years of age (elderly) and 15–64 years of age, although the focus here is on the elderly. The study also examined differences in the associations between cities.

## Materials and Methods

### Data collection

Daily hospital and pollution data were collected for the years 1998 through 2001 in five of the largest cities in Australia (Brisbane, Canberra, Melbourne, Perth, Sydney) and two cities in New Zealand (Auckland, Christchurch). In 2001, these cities covered 53% of the Australian population and 44% of the New Zealand population.

### Cardiovascular health data and air pollution data

Health data were collected for all cardiovascular emergency hospital admissions from state government health departments in Australia and the New Zealand Health Information Service (Ministry of Health). The cardiovascular disease categories used in the study are shown in Table 1, and summary statistics and demography for each city are shown in Table 2.

The pollutants considered were particulate matter < 2.5 μm in diameter (PM_2.5_) and <10 μm in diameter (PM_10_) in micrograms per cubic meter; nitrogen dioxide in parts per billion; carbon monoxide in parts per million; and ozone in parts per billion. Tapered element oscillating microbalance (TEOM) air samplers provided the PM data. Daily pollutant levels were calculated by averaging over a network of monitors in each city. The summary statistics for air pollutants and weather are shown in Table 3.

CO and NO_2_ were the only pollutants monitored in all seven cities on a daily basis. For PM_2.5_, daily measurements were available in four of the Australian cities: Brisbane, Melbourne, Perth, and Sydney. PM_10_ was measured on a daily basis in these four cities and in Christchurch.

### Statistical methods

We used the time-stratified case–crossover method to find associations between pollutants and daily counts of hospital admissions (Janes et al. 2005). Controls were chosen from strata of length 28 days; days either side of the case day were excluded to reduce the correlation between case and control exposure. The method controlled for long-term trend, seasonal changes, and respiratory epidemics by design. Using covariates, there were additional controls for temperature, current minus previous day’s temperature, relative humidity, pressure, extremes of hot and cold (coldest and warmest 1% of days), day of the week, public holiday (yes/no), and day after a public holiday(s) (yes/no). Rainfall was also included in some investigational models.

The pollutant exposure was the average of the current and previous day. Changes in admissions are shown for a one interquartile range (IQR) increase in pollutant, using the mean IQR across cities. This makes the increases from different pollutants more comparable. An IQR increase can be thought of as the difference between a moderately good day and a moderately bad day. The IQRs were 3.8 μg/m^3^ for 24-hr PM_2.5_, 7.5 μg/m3 for 24-hr PM_10_, 5.1 ppb for 24-hr NO_2_, 0.9 ppm for 8-hr CO, and 8.8 ppb for 8-hr O_3_.

To estimate the average effect for all cities, we combined the estimates across cities using a random effects meta-analysis (Normand 1999) and quantified the differences (heterogeneity) between cities using the *I*^2^ statistic (Higgins and Thompson 2002). *I* ^2^ values > 80% indicate that differences between cities are high; > 50%, notable; > 20%, mild; and < 20%, small. To test whether one city had an undue influence on the meta-analysis, we used a leave-one-city-out sensitivity analysis (Normand 1999).

We examined differences in the increases between cities using a hierarchical model to incorporate variables that differ between cities and therefore could modify the results (effect modifiers) (Dominici et al. 2002a). The increases in admissions in each city were regressed against potential city-level effect modifiers such as average pollutant level, temperature, and percentage of the population ≥ 65 years of age. Differences were examined only where there was notable heterogeneity (defined by *I*^2^ > 50%).

When a health outcome showed a significant association with more than one pollutant, we ran a multipollutant model using a matched case–crossover approach (Schwartz 2004). Matching is a traditional approach to control for potential confounding in epidemiology. With control days that are both close in time to the case day and also matched on the level of another pollutant, the effect estimate cannot be confounded by the other pollutant. Matched control days were defined as 24-hr PM_2.5_ within 2 μg/m^3^, 24-hr PM_10_ within 3 μg/m3, 24-hr NO_2_ within 1 ppb, 24-hr CO within 0.5 ppm, and temperature within 1°C.

All analyses were conducted using SAS software (SAS Institute Inc. 2001).

In the absence of an *a priori* opinion of which pollutants were important to health, we used a statistical significance level of 5%, with no correction for multiple comparisons. Although this increased the chances of finding spurious associations, it reduced the chances of missing any important associations during this early stage of investigation of the effects of air pollution in Australia and New Zealand.

In this study we used monitoring data provided by the relevant monitoring agency in each city. The data sets have been used without extensive analysis or corrections beyond the basic quality control needed to ensure data validity for the case–crossover analysis. Some data sets were not fully used (e.g., the PM_10_ data from Auckland) because they did not fully meet the strict requirements of the study but are still regarded as valid data sets for the purposes for which they were gathered.

## Results

The associations between pollutants and cardiovascular hospital admissions are shown in Table 4. In the elderly, significant associations were found between the pollutants CO, NO_2_, and PM and five categories of cardiovascular disease admissions. Arrhythmia showed no associations in the elderly but did in the 15- to 64-year age group. Stroke was the only disease category to show no associations in either age group. O_3_ was the only pollutant to show no associations.

In elderly admissions, the two largest statistically significant increases were for cardiac failure, with a 6.9% increase for a 5.1-ppb unit increase in NO_2_ and a 6.0% increase for a 0.9-ppm increase in CO.

**Figure 1:**
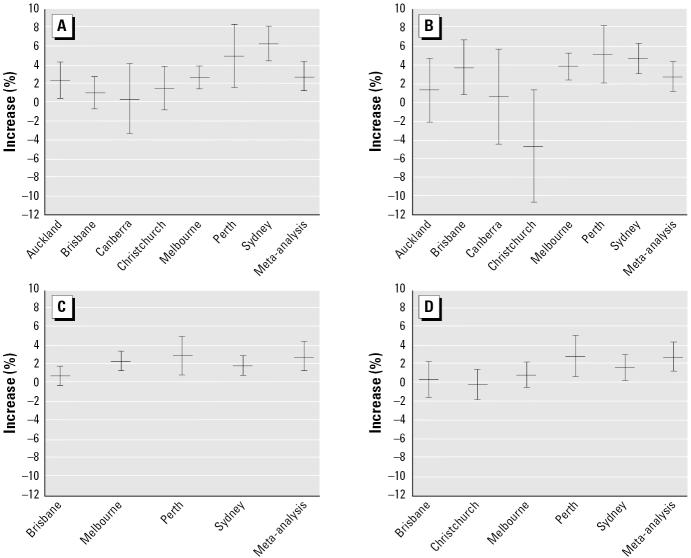
Estimated increases (mean and 95% CI) for cardiac admissions in the elderly by city for four pollutants (average lag, 0–1; one IQR increase). (*A*) Maximum 8-hr CO. (*B*) Average 24-hr NO_2_. (*C*) Average 24-hr PM_2.5_. (*D*) Average 24-hr PM_10_.

For the elderly age group, the relative risks for all cardiac admissions associated with CO, NO_2_, PM_2.5_, and PM_10_ are shown for each city and the meta-analysis in Figure 1, which highlights some of the differences in risk among the cities. This heterogeneity is quantified by the *I*^2^ statistics in Table 4. The *I*^2^ statistics indicate that more than half of the results had small heterogeneity. Notable heterogeneity was more often observed in the elderly group.

Table 5 shows a much reduced *I*^2^ when Sydney was left out for the association between CO and cardiac admissions. Figure 1A shows that the association in Sydney was much larger than in the other cities. The association was also larger in Perth, but the confidence intervals (CIs) were wider. Table 5 and Figure 1B show that when Christchurch was left out, the association between NO_2_ and cardiac admissions was similar for the remaining cities.

Statistically significant effect modifiers were found only for associations with PM_2.5_. For cardiac admissions, there was a greater association with PM_2.5_ in cities with less humidity. For cardiac failure, there was a greater association with PM_2.5_ in cities with higher pressure and a greater percentage of elderly.

Multipollutant results using a matched case–crossover analysis are shown in Table 6. None of the estimated increases changed greatly when cases and controls were matched on temperature. The estimated increase due to NO_2_ fell greatly when cases and controls were matched on CO.

## Discussion

### Cardiovascular admissions in the elderly

This study found many associations between air pollution and cardiovascular admissions in cities in Australia and New Zealand. For every condition but arrhythmia, the increases in hospital admissions were greater in the elderly than in the younger age group (Table 4), most likely because the elderly are a frailer population with probable preexisting heart problems. The frailty of the elderly is also the most likely reason that they did not show increases in arrhythmia. Arrhythmia and cardiac failure are related conditions because atrial fibrillation is a type of arrhythmia and may precipitate cardiac failure in elderly people (Cowie et al. 1999). Hence, exposure to NO_2_ and CO that led to arrhythmia in the younger age group led to the more serious condition of cardiac failure in the elderly.

We found associations at concentrations below normal air quality health guidelines (Table 3). This suggests that current air pollution guidelines need to be revised. There is good reason to believe that lowering air pollution levels would lead to improvements in cardiovascular health.

This results presented here are based on statistically significant findings. Although this is not ideal practice, there is limited space in this article; a complete set of results will be available in a forthcoming report (Expansion of the Multi-City Mortality and Morbidity Study, National Environment Protection Council). A non-statistically significant association does not, of course, mean that a relationship does not exist. This is particularly important for those admissions with smaller numbers of events and hence less power (e.g., stroke in the younger age group).

### Differences among cities

Differences in the associations between cities in this study were mostly not notable (*I*^2^ < 50%). This suggests that the relationship between exposure and disease was often similar. There was more notable heterogeneity in the elderly population, which is partly due to the greater size of the associations in this age group.

In an attempt to explain the notable heterogeneity, we used effect-modifier analyses. However, we found effect modifiers only for associations with PM_2.5_. The effect of PM_2.5_ in Australian cities depended on the percentage of the elderly and average weather conditions. Less average humidity and higher average pressure led to a greater association. To investigate these modifications further, we reran the case–crossover models in each city including an interaction term for 24-hr PM_2.5_ and rainfall (results not shown). Higher rainfall led to a smaller association between cardiovascular admissions and PM_2.5_ in all four cities. This is not surprising, considering that rainfall is a primary removal mechanism for PM_10_ and PM_2.5_, but less so for gaseous pollutants.

### Comparison with three other large studies

The aim and design of this study were similar to those of three other large studies: the APHEA2 study of eight cities in Europe (Le Tertre et al. 2002), NMMAPS with 14 U.S. cities (Samet et al. 2000), and a Canadian study of 10 cities (Burnett et al. 1997b). The results here for PM pollution in terms of elderly cardiac admissions are similar to those found in APHEA2 and NMMAPS, and congestive heart failure in the Canadian study. For example, we found the mean increase for cardiac admissions for the 15–64-year age group to be less than half that in the older group, a result similar to that of the APHEA2 study. The APHEA2 study also found that the heterogeneity in total cardiac admissions (all ages) was related to the percentage of elderly. However, the results for the confounding effects on PM associations by including CO are different (but PM was not monitored in every city here).

A difference between this study and the three large multicity studies is that emission sources for PM, and therefore the PM composition, differ. For example, Chan et al. (1999) found significantly higher contributions from sea salt and nonanthropogenic crustal sources for both PM_2.5_ and PM_10_ in Brisbane than in overseas cities.

Another important difference from the other studies is in the statistical methods used here. The NMMAPS and APHEA2 studies used generalized additive models, in which confounding was estimated by adding the copollutant into the model. In the APHEA2 study, the PM_10_ associations were significantly reduced in the multipollutant models by the inclusion of NO_2_ (as found here) and slightly (but becoming statistically insignificant) for CO. However, using black smoke to estimate the PM impacts showed no confounding by CO and much less by NO_2_. There was no significant confounding of the PM associations by CO or NO_2_ found in NMMAPS.

### Addressing confounding between pollutants

Instead of using a multipollutant model, we controlled for confounding by matching in the case–crossover analysis. For elderly hospital admissions, the CO associations remained of a similar size when matched with NO_2_. Conversely, the NO_2_ became smaller when matched with CO (Table 6).

Matching was also used to control for the important confounder of temperature. The results changed little when matched on temperature, strongly suggesting that the association between air pollution and cardiovascular disease is not confounded by temperature.

### Is outdoor air pollution a good indicator of exposure?

A problem in interpreting the results from this study is that it used outdoor air pollution concentrations measured at fixed-point monitors (ambient concentrations), whereas people spend most of their time indoors. Recent studies in Baltimore, Maryland (Sarnat et al. 2001) and Boston, Massachusetts (Sarnat et al. 2005) indicate that such ambient concentrations may be poor surrogates for actual exposure to air pollution, especially in winter when buildings are more sealed. However, winters in Australia are mild, meaning that people will likely spend more time outdoors and that houses are designed to lose heat rather than trap it. Hence, exposure to the air may be high all year round in Australia (winter exposure in New Zealand may be more similar to that in Baltimore). A similar conclusion was drawn by a study of the effects of cold temperatures on cardiovascular disease (Barnett et al. 2005). In that study, regions with mild winters showed greater increases in cold-related cardiovascular events than did regions with usually cold winters.

The study in Baltimore also found that ambient concentrations for CO and NO_2_ were often better surrogates for actual exposure to PM than to CO and NO_2_, especially in summer (Sarnat et al. 2001). However, the more recent Boston study did note that there were some correlations between ambient concentrations and actual exposure to these gases in summer (Sarnat et al. 2005). Outdoor concentrations for pollutants such as NO_2_, CO, and PM often arise from the same combustion emissions sources, such as motor exhausts.

### CO as a marker for pollution sources

There is evidence that air pollutants (NO_2_, CO) may trigger fibrillation in people with a history of serious arrhythmia (Peters et al. 2000). The effect of CO on cardiovascular disease is well known, with CO replacing oxygen in the blood stream, but at the low CO concentrations prevailing in the cities under study, it cannot be simply assumed that if CO is the “cause” of any effects found here, it is due to this mechanism. The associations found for CO, NO_2_, and PM are not additive, but probably refer to the impacts of a similar pollutant “mix.” Given that the CO associations show the least change when matched with the other pollutants (Table 6), this indicates that the air pollutant mixture arising from emission sources dominating the CO emissions (usually human combustion sources) is the primary cause of the association, not the effect of CO itself.

## Conclusions

For both Australian and New Zealand cities, the results show that increases in outdoor concentrations of CO, NO_2_, and PM have significant associations with increases in cardiovascular admissions for adults, especially the elderly (≥ 65 years of age). Associations were found at concentrations below normal air quality health guidelines. There were significant associations between air pollution and arrhythmia admissions in the younger age group, which were not apparent for the elderly. For the elderly, there were significant associations between air pollution increases and increases in hospital admissions for ischemic heart disease and myocardial infarction, and these were not apparent for the younger group. Atrial fibrillation can precipitate cardiac failure, especially in the elderly, and a significant relationship has been identified here in the adult age group (15–64 years) between increases in hospital admissions for arrhythmia and increases in air pollution.

The associations for NO_2_ appear to be stronger in Australian than in New Zealand cities, whereas those of CO are similar for cities in both countries. In Australian cities, PM_10_ and PM_2.5_ had a similar association, apart from that for arrhythmia. These PM_2.5_ associations differed among cities due to different climate conditions for humidity (the lower the humidity, the greater the association).

It is difficult to separate the associations for different pollutants because there are common emission sources for CO, NO_2_, and PM (e.g., motor vehicle exhausts). Also, outdoor concentrations are often not good surrogates for actual exposure, with outdoor levels for the gases sometimes being good surrogates for actual exposure to PM, especially in summer.

## Figures and Tables

**Table 1:** Cardiovascular disease categories and *International Classification of Disease* (ICD) codes.

Disease category	ICD-9	ICD-10
Arrhythmia	427	I46–I49
Cardiac disease	390–429	I00–I52, I97.0, I97.1, I98.1
Cardiac failure	428	I50
Ischemic heart disease	410–413	I20, I21, I22, I24, I25.2
Myocardial infarction	410	I21, I22
Stroke	430–438	I60–I66, I67 (excluding I67.0, I67.3), I68 (excluding I68.0), I69, G45 (excluding G45.3), G46
Total cardiovascular disease	390–459	I00–I99 (excluding I67.3, I68.0, I88, I97.8, I97.9, I98.0), G45 (excluding G45.3), G46, M30, M31, R58

ICD-9, 9th Revision, used January–June 1998; ICD-10, 10th Revision, used July 1998–December 2001.

**Table 2:** Summary statistics for demographic and hospital admission rates per million people (1998–2001).

	Auckland	Brisbane	Canberra	Christchurch	Melbourne	Perth	Sydney
Demographic data							
Total population	1,158,891	1,627,535	311,518	316,224	3,366,542	1,339,993	3,997,321
Percentage of population > 65 years	10.0	11.0	8.3	13.7	12.1	11.3	11.9
Daily hospital admissions [mean (range)]							
Cardiovascular							
15–64 years	11.5 (3–31)	9.3 (2–19)	17.6 (0–67)	10.2 (0–32)	7.7 (2–14)	7.9 (1–17)	7.7 (3–14)
≥65 years	18.1 (4–35)	18.6 (8–33)	20.0 (0–61)	26.2 (0–60)	16.3 (8–28)	18.8 (6–35)	15.5 (7–26)
Cardiac							
15–64 years	8.6 (1–24)	7.5 (1–17)	10.5 (0–42)	7.3 (0–28)	5.5 (1–11)	6.0 (0–14)	5.9 (2–11)
≥65 years	12.8 (2–27)	14.0 (5–29)	14.1 (0–45)	18.1 (0–44)	11.5 (5–21)	13.7 (3–27)	11.1 (5–20)
Ischemic heart disease							
15–64 years	4.5 (0–14)	4.5 (0–11)	4.8 (0–26)	4.5 (0–19)	3.2 (1–6)	3.4 (0–9)	3.1 (0–7)
≥65 years	6.5 (0–18)	7.6 (1–18)	6.1 (0–26)	10.4 (0–35)	5.7 (2–11)	6.7 (1–15)	4.9 (2–10)
Stroke							
15–64 years	1.6 (0–7)	0.9 (0–6)	1.0 (0–10)	1.8 (0–13)	1.1 (0–3)	1.0 (0–5)	1.0 (0–3)
≥65 years	3.5 (0–10)	3.2 (0–9)	2.6 (0–16)	5.5 (0–22)	3.5 (1–10)	3.4 (0–9)	3.1 (1–7)
Arrhythmia							
15–64 years	2.0 (0–9)	1.3 (0–6)	2.0 (0–19)	1.3 (0–13)	1.0 (0–4)	1.1 (0–6)	1.2 (0–4)
≥65 years	2.5 (0–9)	2.1 (0–7)	2.5 (0–16)	2.7 (0–16)	1.8 (0–5)	2.2 (0–7)	1.9 (0–5)
Cardiac failure							
15–64 years	0.8 (0–5)	0.5 (0–3)	0.4 (0–6)	0.4 (0–9)	0.5 (0–3)	0.5 (0–4)	0.4 (0–2)
≥65 years	2.7 (0–10)	3.2 (0–10)	2.3 (0–13)	3.7 (0–22)	3.3 (1–7)	3.7 (0–12)	2.9 (0–9)
Myocardial infarction							
15–64 years	1.5 (0–7)	1.4 (0–5)	1.2 (0–10)	1.8 (0–13)	1.2 (0–4)	1.3 (0–5)	1.1 (0–3)
≥65 years	2.3 (0–9)	2.4 (0–7)	1.4 (0–13)	4.7 (0–25)	1.9 (0–6)	2.4 (0–9)	1.7 (0–4)

**Table 3:** Summary statistics for daily air pollutant and weather data (1998–2001).

	Auckland	Brisbane	Canberra	Christchurch	Melbourne	Perth	Sydney
Daily pollutant levels [mean (range)]							
24-hr PM_2.5_ (μg/m^3^)	11.0^a^ (2.1–37.6)	9.7 (3.2–122.8)	—	—	8.9 (2.8–43.3)	8.1 (1.7–29.3)	9.4 (2.4–82.1)
No. of monitors	1	1	0	0	2	2	3
24-hr PM_10_ (μg/m^3^)	18.8^a^ (3.2–101.4)	16.5 (3.8–50.2)	—	20.6 (1.3–156.3)	16.6 (3.1–71.1)	16.5 (4.4–68.9)	16.6 (3.7–104.7)
No. of monitors	6	4	0	2	4	1	11
1-hr NO_2_ (ppb)	19.1 (4.2–86.3)	17.3 (4–44.1)	17.9 (0–53.7)	15.7 (1.2–54.6)	23.2 (4.4–62.5)	21.3 (4.4–48)	22.6 (5.2–51.4)
24-hr NO_2_ (ppb)	10.2 (1.7–28.9)	7.6 (1.4–19.1)	7.0 (0–22.5)	7.1 (0.2–24.5)	11.7 (2–29.5)	9.0 (2–23.3)	11.5 (2.5–24.5)
No. of monitors	2	7	1	1	8	5	13
8-hr CO (ppb)	2.1 (0.2–7.9)	1.7 (0–7)	0.9 (0–5.8)	0.5 (0–5.4)	1.0 (0.1–8)	1.0 (0.1–4)	0.8 (0–4.5)
No. of monitors	3	1	1	2	3	3	4
1-hr O_3_ (ppb)	—	31.5 (7–92.3)	—	—	23.8 (1.7–85.4)	33.6 (13–85)	31.7 (3.2–126.7)
4-hr O_3_ (ppb)	—	28.9 (5.4–75.2)	—	—	21.8 (1.3–73.1)	31.3 (10.6–72.8)	28.9 (2.2–105.1)
8-hr O_3_ (ppb)	—	25.5 (3.7–58.4)	—	—	19.0 (0.8–63)	28.5 (8–64)	24.9 (1.4–86.8)
No. of monitors	0	7	0	0	8	3	12
Weather							
Temperature (°C)	15.7 (6.3–24.1)	20.0 (9.5–30.4)	13.7 (1–28)	11.6 (0–27.2)	15.3 (5.9–31.8)	18.2 (8.2–32.3)	17.8 (8.5–30.1)
Relative humidity (%)	79.1 (52.1–100)	72.4 (29.3–96.3)	69.9 (24.1–97)	75.9 (31–99)	68.7 (25.1–95.5)	67.8 (28–98.5)	70.6 (26.3–97.1)
Rain (mm)	3.06 (0–71.6)	2.6 (0–128.9)	1.82 (0–79.8)	1.56 (0–54.8)	1.99 (0–43.07)	2.08 (0–104.0)	2.71 (0–137.1)

a The Auckland region does not run TEOMs but had an extensive Hi-Vol network. The PM data for Auckland were recorded once every 6 days and were not suitable for the case–crossover analysis.

**Table 4:** Significant increases in cardiovascular hospital admissions by age group using a meta-analysis of case–crossover estimates (urban Australia and New Zealand, 1998–2001).

		Elderly (≥65 years)		Adults (15–64 years)	
Disease category	Pollutant (units)	Increase [%^a^ (95% CI)]	*I*^2^ (%)^b^	Increase [%^a^ (95% CI)]	*I*^2^ (%)^b^
Arrhythmia	24-hr NO_2_ (ppb)	0.4 (−1.8 to 2.6)	0	5.1 (2.2 to 8.1)	0
	8-hr CO (ppm)	0.1 (−1.8 to 2.1)	10.8	2.5 (0.1 to 4.9)	5.6
Cardiac	24-hr PM_2.5_ (μg/m^3^)^c^	1.9 (1.0 to 2.7)	55.0	0.6 (−0.2 to 1.4)	0
	24-hr PM_10_ (μg/m^3^)^c^	1.1 (0.2 to 2.0)	32.9	0.3 (−0.8 to 1.3)	0
	24-hr NO_2_ (ppb)	3.4 (1.9 to 4.9)	54.1	2.2 (0.9 to 3.4)	0
	8-hr CO (ppm)	2.8 (1.3 to 4.4)	73.5	1.7 (0.5 to 2.9)	24.7
Cardiac failure	24-hr PM_2.5_ (μg/m^3^)^c^	3.6 (1.8 to 5.4)	58.6	3.0 (−0.1 to 6.1)	0
	24-hr PM_10_ (μg/m^3^)^c^	3.4 (2.1 to 4.7)	0	2.1 (−1.7 to 6.1)	0
	24-hr NO_2_ (ppb)	6.9 (2.2 to 11.8)	61.3	4.6 (0.1 to 9.3)	0
	8-hr CO (ppm)	6.0 (3.5 to 8.5)	61.6	4.2 (0.6 to 7.8)	0
Ischemic heart disease	24-hr PM_2.5_ (μg/m^3^)^c^	1.6 (0.7 to 2.4)	3.6	0.2 (−0.9 to 1.3)	0
	24-hr NO_2_ (ppb)	2.5 (1.0 to 4.1)	19.7	0.7 (−1.0 to 2.4)	0
	8-hr CO (ppm)	2.3 (0.9 to 3.8)	35.9	1.6 (−0.6 to 3.9)	53.5
Myocardial infarction	24-hr PM_2.5_ (μg/m^3^)^c^	2.7 (1.3 to 4.2)	0	1.2 (−0.6 to 3.1)	0
	24-hr NO_2_ (ppb)	4.4 (1.0 to 8.0)	38.2	1.7 (−1.1 to 4.7)	0
	8-hr CO (ppm)	2.9 (0.8 to 4.9)	21.3	1.8 (−0.7 to 4.3)	9.2
Total cardiovascular	24-hr PM_2.5_ (μg/m^3^)^c^	1.3 (0.6 to 2.0)	51.9	0.2 (−0.5 to 0.9)	0
	24-hr NO_2_ (ppb)	3.0 (2.1 to 3.9)	18.4	1.7 (0.6 to 2.8)	0
	8-hr CO (ppm)	2.2 (0.9 to 3.4)	69.5	1.2 (0.3 to 2.1)	6.6

CI, confidence interval.

a Percent increase in admissions for an IQR increase in pollutant using the average over the current and previous day.

b *I*^2^ is the percentage of total variation in the estimated increase that is due to heterogeneity between cities.

c PM_2.5_ was measured on a daily basis only in Brisbane, Melbourne, Perth, and Sydney; and PM_10_, in these cities and Christchurch.

**Table 5:** Significant increases in cardiac hospital admissions for the elderly age group for the cities: results from the leave-one-out sensitivity analysis.

	8-hr CO		24-hr NO_2_		24-hr PM_2.5_	
City left out	Increase [%^a^ (95% CI)]	*I*^2^ (%)^b^	Increase [%^a^ (95% CI)]	*I*^2^ (%)^b^	Increase [%^a^ (95% CI)]	*I*^2^ (%)^b^
Auckland	2.9 (1.1–4.7)	77.7	3.6 (2.1–5.2)	54.1	[Table-fn tfn7-ehp0114-001018]—	[Table-fn tfn7-ehp0114-001018]—
Brisbane	3.2 (1.5–4.9)	71.5	2.8 (0.5–5.1)	61.7	2.2 (1.6–2.9)	0.0
Canberra	3.1 (1.5–4.7)	76.1	3.5 (2.0–5.1)	56.8	[Table-fn tfn7-ehp0114-001018]—	[Table-fn tfn7-ehp0114-001018]—
Christchurch	3.0 (1.3–4.8)	76.5	4.0 (3.0–5.0)	7.4	[Table-fn tfn7-ehp0114-001018]—	[Table-fn tfn7-ehp0114-001018]—
Melbourne	2.8 (1.0–4.7)	77.8	2.6 (0.2–5.2)	61.6	1.6 (0.6–2.8)	57.6
Perth	2.6 (0.9–4.2)	76.3	3.0 (1.3–4.7)	59.2	1.7 (0.7–2.6)	61.4
Sydney	2.2 (1.2–3.1)	21.7	2.8 (1.0–4.7)	55.0	1.9 (0.6–3.2)	69.7
Both New Zealand cities	3.2 (1.0–5.3)	80.8	4.2 (3.3–5.2)	0.0	1.9 (1.0–2.7)	55.0

— , not collected.

a Percent increase in admissions for an IQR increase in pollutant using the average over the current and previous day.

b *I*^2^ is the percentage of total variation in the estimated increase that is due to heterogeneity between cities.

**Table 6:** Multipollutant models: statistically significant increases in cardiac hospital admissions and increases after matching for other exposures.

Age group/single pollutant	Matched exposure	Increase [%^a^ (95% CI)]
Cardiac admissions (15–64 years of age)		
24-hr average NO_2_	8-hr average CO (maximum)	−0.0 (−1.6 to 1.5)
	Average temperature	2.4 (0.9–4.0)
	Unmatched	2.2 (0.9–3.4)
8-hr average CO (max)	24-hr average NO_2_	2.4 (−1.4 to 6.3)
	Average temperature	1.9 (0.6–3.3)
	Unmatched	1.7 (0.5–2.9)
Cardiac admissions (≥65 years of age)		
24-hr average NO_2_	8-hr average CO (maximum)	0.6 (−1.7 to 3.0)
	Average temperature	3.5 (1.7–5.4)
	Unmatched	3.4 (1.9–4.9)
		0.4 (−1.7 to 2.5)
24-hr average PM_2.5_^b^	24-hr average NO_2_	
	8-hr average CO (maximum)	1.2 (0.0–2.4)
	Average temperature	1.8 (0.6–3.0)
	Unmatched	1.9 (1.0–2.7)
8-hr average CO (maximum)	24-hr average NO_2_	2.8 (0.2–5.4)
	Average temperature	2.6 (0.9–4.4)
	Unmatched	2.8 (1.3–4.4)

a Percent increase in admissions for an IQR increase in pollutant using the average over the current and previous day.

b PM_2.5_ was measured on a daily basis only in Brisbane, Melbourne, Perth, and Sydney; and PM_10_, in these cities and Christchurch.
